# Lipopeptide biosynthesis in *Pseudomonas fluorescens* is regulated by the protease complex ClpAP

**DOI:** 10.1186/s12866-015-0367-y

**Published:** 2015-02-14

**Authors:** Chunxu Song, Gustav Sundqvist, Erik Malm, Irene de Bruijn, Aundy Kumar, Judith van de Mortel, Vincent Bulone, Jos M Raaijmakers

**Affiliations:** Laboratory of Phytopathology, Wageningen University, 6708 PB Wageningen, the Netherlands; Department of Microbial Ecology, Netherlands Institute of Ecology, Droevendaalsesteeg 10, 6708 PB Wageningen, the Netherlands; Division of Glycoscience, Royal Institute of Technology (KTH), AlbaNova University Centre, SE-106 91 Stockholm, Sweden; Division of Plant Pathology, Indian Agricultural Research Institute, New Delhi, 110012 India; Current address: HAS University of Applied Sciences, 5911 KJ Venlo, the Netherlands

## Abstract

**Background:**

Lipopeptides (LP) are structurally diverse compounds with potent surfactant and broad-spectrum antibiotic activities. In *Pseudomonas* and other bacterial genera, LP biosynthesis is governed by large multimodular nonribosomal peptide synthetases (NRPS). To date, relatively little is known about the regulatory genetic network of LP biosynthesis.

**Results:**

This study provides evidence that the chaperone ClpA, together with the serine protease ClpP, regulates the biosynthesis of the LP massetolide in *Pseudomonas fluorescens* SS101. Whole-genome transcriptome analyses of *clpA* and *clpP* mutants showed their involvement in the transcription of the NRPS genes *massABC* and the transcriptional regulator *massAR.* In addition, transcription of genes associated with cell wall and membrane biogenesis, energy production and conversion, amino acid transport and metabolism, and pilus assembly were altered by mutations in *clpA* and *clpP*. Proteome analysis allowed the identification of additional cellular changes associated to *clpA* and *clpP* mutations. The expression of proteins of the citrate cycle and the heat shock proteins DnaK and DnaJ were particularly affected. Combined with previous findings, these results suggest that the ClpAP complex regulates massetolide biosynthesis via the pathway-specific, LuxR-type regulator MassAR, the heat shock proteins DnaK and DnaJ, and proteins of the TCA cycle.

**Conclusions:**

Combining transcriptome and proteome analyses provided new insights into the regulation of LP biosynthesis in *P. fluorescens* and led to the identification of specific missing links in the regulatory pathways.

**Electronic supplementary material:**

The online version of this article (doi:10.1186/s12866-015-0367-y) contains supplementary material, which is available to authorized users.

## Background

Lipopeptides (LPs) are biosurfactants produced by a variety of bacterial genera, including *Pseudomonas* and *Bacillus *[1,2]. LPs are composed of an (cyclic) oligopeptide moiety linked to a fatty acid tail [1]. In beneficial *Pseudomonas* strains, LPs play a role in colonization of seeds [3] and roots [4], in defense against competing microorganisms and predatory protozoa [5], and in swarming motility and biofilm formation [6]. LP biosynthesis is governed by large multi-modular nonribosomal peptide synthetases (NRPS) via a thiotemplate process [1,7]. Compared to our understanding of LP biosynthesis, relatively little is known about the genetic networks involved in the perception of external signals and the signal transduction pathways that drive transcription of the LP biosynthesis genes. Here we focus on the regulation of LP biosynthesis in the plant growth-promoting rhizobacterium *Pseudomonas fluorescens* SS101. Strain SS101 produces the LP massetolide A, a 9-amino-acid cyclic peptide linked to 3-hydroxydecanoic acid [8,9]. Massetolide A is produced in the early exponential growth phase and is essential for swarming motility and biofilm formation of strain SS101 [8]. Its biosynthesis is governed by three NRPS genes, designated *massA, massB*, and *massC* [8].

To identify the genetic networks underlying regulation of massetolide biosynthesis, *P. fluorescens* strain SS101 was subjected to random mutagenesis. Screening of a library of approximately 7,500 random plasposon mutants resulted in the identification of four new regulatory genes, namely *phgdh*, *dnaK*, *prtR* and *clpA* [10]. In this recent study, we focused our functional analyses on *phgdh*, *dnaK* and *prtR*, but not on *clpA*. Independently from this work, *clpP* had been previously identified as a regulator of massetolide biosynthesis in *P. fluorescens* SS101 [11]. Hence, the aims of the present study were to i) study the role of ClpA in regulation of massetolide biosynthesis, and ii) analyse the ClpA regulon at the transcriptional and proteome level in order to narrow down the role of ClpP in regulating massetolide biosynthesis.

The ATP-dependent serine protease ClpP is highly conserved in eubacteria [12] and has diverse functions, including intracellular proteolysis. ClpP associates with different ATPases that either recognize protein substrates directly or, alternatively, interact with substrates via so-called adaptor proteins [13]. Substrates are then unfolded and translocated to the proteolytic chamber of the ClpP protease [14]. ClpP consists of two heptameric rings that form a barrel-shaped proteolytic core with the active sites hidden in an interior chamber [15]. The ATPases of ClpP that have been studied in detail in various bacterial genera include ClpX, ClpB, HslU and ClpA [16,17]. In strain SS101, site-directed mutagenesis of *clpX* did not affect massetolide biosynthesis [11], suggesting that ClpX does not act as the chaperone of ClpP in the regulation of massetolide biosynthesis. Therefore, the focus of our present study is on the role of the ClpAP complex in the regulation of massetolide biosynthesis. ClpA is formed as a hexameric chaperone ring complex and selects the target proteins for ClpP to degrade based on the N-end rule [18]. Either misfolded or specifically tagged proteins are targeted by ClpA [19]. To unravel the cellular substrates of the ClpAP complex in *E.coli*, a proteomics approach [20] was adopted which revealed that several proteins involved in metabolism and energy production, cell motility and transport are potential cellular targets. In our study, we combined transcriptomic and proteomic analyses for both *clpA* and *clpP* mutants to identify putative substrates of the ClpAP complex with the ultimate goal to further elucidate the genetic regulation of massetolide biosynthesis in *P. fluorescens*.

## Results and discussion

### Role of *clpA* in lipopeptide biosynthesis in *P. fluorescens* SS101

**Figure 1:**
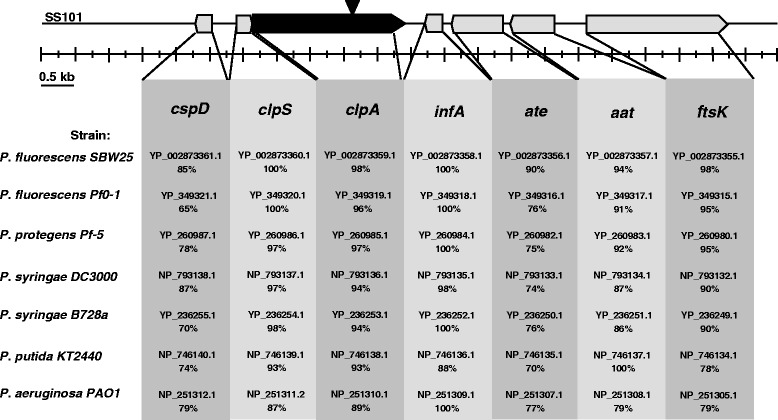
**Genomic organization of** ***clpA*** **and flanking genes in** ***P. fluorescens*** **SS101.** The *clpA* gene (PflSS101_ 3193) and flanking genes in *P. fluorescens* SS101 and the percentages of amino acid identity with their corresponding homologues in other *Pseudomonas* species and strains are indicated. The triangle indicates the position of the plasposon insertion in the *clpA* gene. Abbreviations: *cspD*: cold shock domain protein; *clpS*: ATP-dependent Clp protease adaptor protein; *clpA*: ATP-dependent Clp protease ATP-binding subunit; *infA*: translation initiation factor IF-1; *ate*: putative arginyl-tRNA-protein transferase; *aat*: leucyl/phenylalanyl-tRNA-protein transferase; *ftsK*: DNA translocase.

**Figure 2:**
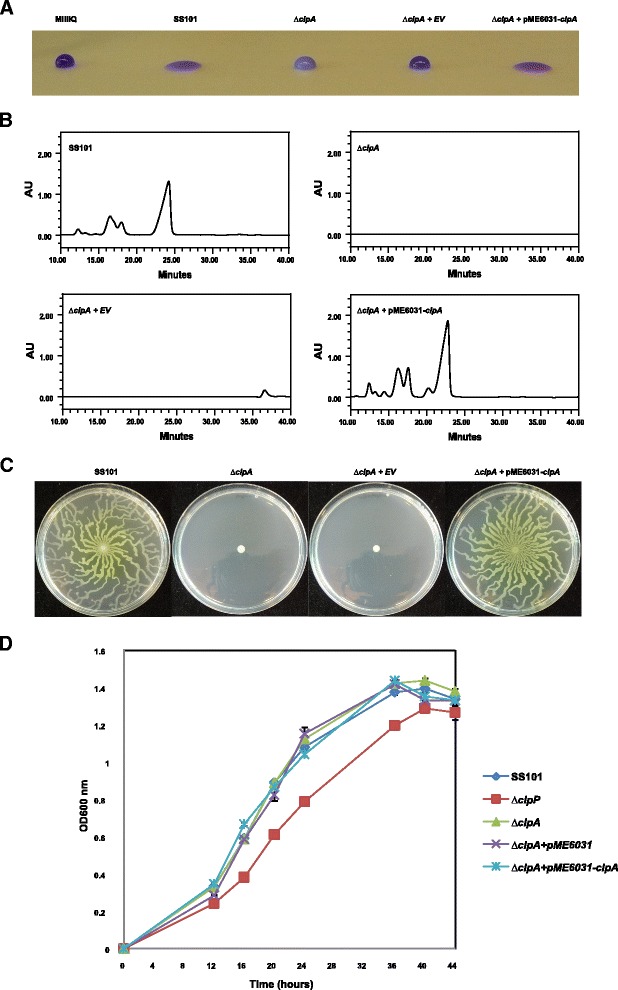
**Phenotypic and chemical analyses of** ***P. fluorescens*** **strain SS101, and its** ***clpA*** **mutant. (A)** Drop collapse assay with cell suspensions of wild-type strain SS101, *clpA* plasposon mutant, *clpA* mutant + pME6031 (empty vector control) and *clpA* mutant + pME6031-*clpA*. Bacterial cultures grown for 2 days at 25°C on KB agar plates were suspended in sterile water to a final density of 1x10^10^ cells/ml. 10-μl droplets were spotted on parafilm and crystal violet was added to the droplets to facilitate visual assessment. A flat droplet is a highly reliable proxy for the production of the surface-active lipopeptide massetolide A. **(B)** RP-HPLC chromatograms of cell-free culture extracts of the wild-type strain SS101, *clpA* plasposon mutant, *clpA* + pME6031 (empty vector control) and *clpA* + pME6031-*clpA* as described in panel A. The wild-type strain SS101 produces massetolide A (retention time of approximately 23–25 min) and various other derivatives of massetolide A (minor peaks with retention times ranging from 12 to 18 min) which differ from massetolide A in the amino acid composition of the peptide moiety. **(C)** Swarming motility of the wild-type strain SS101, *clpA* plasposon mutant, *clpA* mutant + pME6031 (empty vector control) and *clpA* mutant + pME6031-*clpA* on soft (0.6% wt/vol) agar plates. Five microliter (1×10^10^ cells/ml) of washed cells from overnight cultures was spot-inoculated in the center of a soft agar plate and incubated for 48–72 h at 25°C. **(D)** Growth of the wild-type SS101 strain, *clpA* plasposon mutant, *clpA* mutant + pME6031 (empty vector control), *clpA* mutant + pME6031-*clpA* and *clpP* site-directed mutagenesis mutant in liquid medium at 25°C. The optical density of the cell cultures was measured spectrophotometrically (600 nm) at different time points. Mean values of four biological replicates are given; the error bars represent the standard error of the mean.

In *P. fluorescens* SS101, the *clpA* gene is 2271 bp with 89 to 98% identity to homologs in other *Pseudomonas* genomes (Figure 1). Based on the drop collapse assay, a mutation in the *clpA* gene abolishes massetolide production (Figure 2A). RP-HPLC analysis confirmed that the *clpA* mutant indeed did not produce detectable levels of massetolide A or its derivatives (Figure 2B). Complementation of the *clpA* mutant with the stable vector pME6031-*clpA* restored massetolide production to wild-type level, whereas the empty-vector control did not (Figure 2B). Massetolide biosynthesis is known to be essential for swarming motility of strain SS101 [8]. The *clpA* mutant was not able to swarm on soft agar (0.6% w/v; Figure 2C) and this phenotype was restored by complementation with pME6031-*clpA* (Figure 2C). In contrast to a mutation in *clpP*, no effects on growth were observed for the *clpA* mutant (Figure 2D). Collectively, these results indicated that *clpA* is required for massetolide biosynthesis in *P. fluorescens* SS101.

### Transcriptome analysis

To further investigate the genetic basis for ClpAP-mediated regulation of massetolide biosynthesis, whole-genome transcriptome analyses were performed for the *clpA* (Additional file 1: Figure S1A) and *clpP* (Additional file 1: Figure S1B) mutants. Given the differences in growth kinetics between the mutants and wild-type SS101 (Figure 2D), cells were harvested in the exponential growth phase (OD_600nm_ = 0.6). In the *clpA* mutant, transcription of 14 and 37 genes increased and decreased, respectively, by at least 2-fold (P_FDR_ < 0.05) (Additional file 2: Table S1). Apart from the massetolide biosynthesis genes, several of the differentially regulated genes were associated with energy production and conversion, amino acid transport and metabolism, cell wall and membrane biogenesis and pilus assembly. Several of the other differentially regulated genes could not be assigned to clusters of orthologous groups (COGs). Two pili gene clusters were significantly down-regulated in the *clpA* mutant. The first was the *csu* gene cluster (PflSS101_3282-3285) which is known to affect biofilm formation in *Acinetobacter baumannii* [21]. The second was the type IVb pili gene cluster PflSS101_0648-0655 and the regulator *pprB* (Additional file 2: Table S1). In *Pseudomonas aeruginosa*, type IVb pili are required for adhesion to abiotic surfaces and to eukaryotic cells [22]. Further experiments will be needed to explore the functions of both pili gene clusters in *P. fluorescens* SS101.

**Figure 3:**
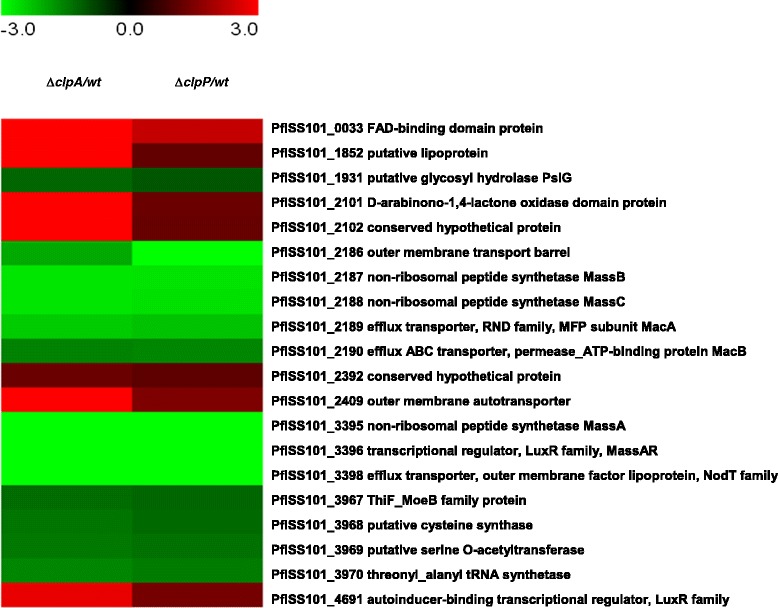
**Heatmaps showing log**_**2**_ **-fold changes in the expression of genes that are differently expressed in the** ***clpA*** **or** ***clpP*** **mutants of** ***Pseudomonas fluorescens*** **SS101.** See supplementary Additional file 2: Tables S1 and S2 for the list of all genes differentially regulated in the *clpA* or *clpP* mutant versus wild-type SS101.

With 195 and 154 genes significantly up and down regulated, respectively, the *clpP* mutation had a much bigger impact, as expected, on the overall gene expression in strain SS101 than a mutation in *clpA* (Additional file 2: Table S2, Additional file 1: Figure S1B). Combining the transcriptome data of the *clpA* and *clpP* mutants revealed that seven and thirteen genes were up and down-regulated, respectively, in both mutants (Figure 3). These include the massetolide biosynthesis genes *massA*, *massB, massC* and their flanking genes consisting of the LuxR-type transcriptional regulator *massAR* and the efflux-associated genes PflSS101_3398, PflSS101_2189 and PflSS101_2190. Among the genes differentially regulated in both *clpA* and *clpP* mutants were also the *thiF_moeB* gene cluster (PflSS101_3967-3970) as well as genes encoding a FAD-binding domain protein (PflSS101_0033) and an auto-inducer-binding LuxR-type transcriptional regulator (PflSS101_4691) (Figure 3). Expression of the previously identified regulatory genes of massetolide biosynthesis, *phgdh*, *dnaK*, and *prtR* [10], was not affected in the *clpA* and *clpP* mutants. This suggests that, at the transcriptional level, *clpAP*-mediated regulation of massetolide biosynthesis operates downstream or operates independently from these other regulatory genes.

### Proteome analysis

Total cell proteomic analyses were performed to further decipher the potential cellular substrates and target proteins of ClpAP (Additional file 1: Figure S2). The culture conditions and ‘harvest’ time of the bacterial cells (OD_600_ = 0.6) were identical to those used in the transcriptome analyses described above. It should be noted that the ClpAP system is a degradative protease thereby complicating the interpretation of proteomics data. While transcriptomics can validly argue that mRNAs (and hence proteins) are up- or down-regulated, the higher abundance of a particular protein in the *clpA* and or *clpP* mutants can also be due to an inherent up- or down-regulation by other modulated pathways. Hence, the proteomics results described below should be interpreted with caution.

### Proteins differentially expressed in the *clpA* mutant or *clpP* mutant

iTRAQ-based proteome analyses allowed the identification of a total of 596 proteins in the *clpA* mutant (Additional file 2: Table S3): 68 proteins were significantly up-regulated (Fold change > 1.2) while 132 were down-regulated (Additional file 2: Table S3). Gap2 (PflSS101_4355), encoding a glyceraldehyde-3-phosphate dehydrogenase, was up-regulated in the *clpA* mutant, which was consistent with the earlier report [20] that reported a similar GapA protein as one of the substrates of ClpAP in *E. coli*. All three protein groups from the ‘intracellular trafficking and secretion’ COG category were up-regulated in the *clpA* mutant, including SecA, SecB, and the Tol-Pal system protein TolB (Additional file 1: Figure S2A, Additional file 2: Table S3).

In line with the findings in *E. coli* [20], we observed that the cell division protein FtsZ and the isocitrate lyase AceA were up-regulated in the *clpP* mutant (Additional file 1: Figure S2B; Additional file 2: Table S4), suggesting that these proteins might be substrates of ClpP in strain SS101. Moreover, we detected five transcriptional regulators and five chaperons that were uniquely up-regulated in the *clpP* mutant (Table 1). One of the up-regulated transcriptional regulators was MvaT (PflSS101_4330), which is known to regulate the biosynthesis of specific secondary metabolites in the rhizobacterium *Pseudomonas protegens* CHA0 [23]. Furthermore, the heat shock proteins DnaK and DnaJ, the chaperonin GroS, GroL and the chaperon HtpG were significantly up-regulated in the *clpP* mutant. Also CheA, a histidine kinase that mediates chemotaxis signaling events in many prokaryotes [24], was 1.49-fold up-regulated, suggesting it may be a substrate of ClpP in strain SS101.

**Table 1:** **Regulator and chaperon proteins differentially expressed in the** ***clpP*** **mutant of** ***Pseudomonas fluorescens*** **SS101**

**Locus**	**Gene**	**Gene description**	**Fold changes in ∆c** ***lpP*** **/SS101**	
PflSS101_1716	*cysB*	HTH-type transcriptional regulator CysB	1.25	up
PflSS101_3936		transcriptional regulator, GntR family	1.35	up
PflSS101_4330	*mvaT*	transcriptional regulator MvaT	1.26	up
PflSS101_4600	*cbrB*	two-component response regulator CbrB	1.50	up
PflSS101_5275	*rnk*	regulator of nucleoside diphosphate kinase	1.65	up
PflSS101_1812	*htpG*	chaperone protein HtpG	1.2	up
PflSS101_4373	*groL*	chaperonin GroL	1.22	up
PflSS101_4374	*groS*	chaperonin GroS	1.32	up
PflSS101_4632	*dnaJ*	chaperone protein DnaJ	1.21	up
PflSS101_4633	*dnaK*	chaperone protein DnaK	1.32	up

### Proteins differentially expressed in both *clpA* and *clpP* mutants

**Figure 4:**
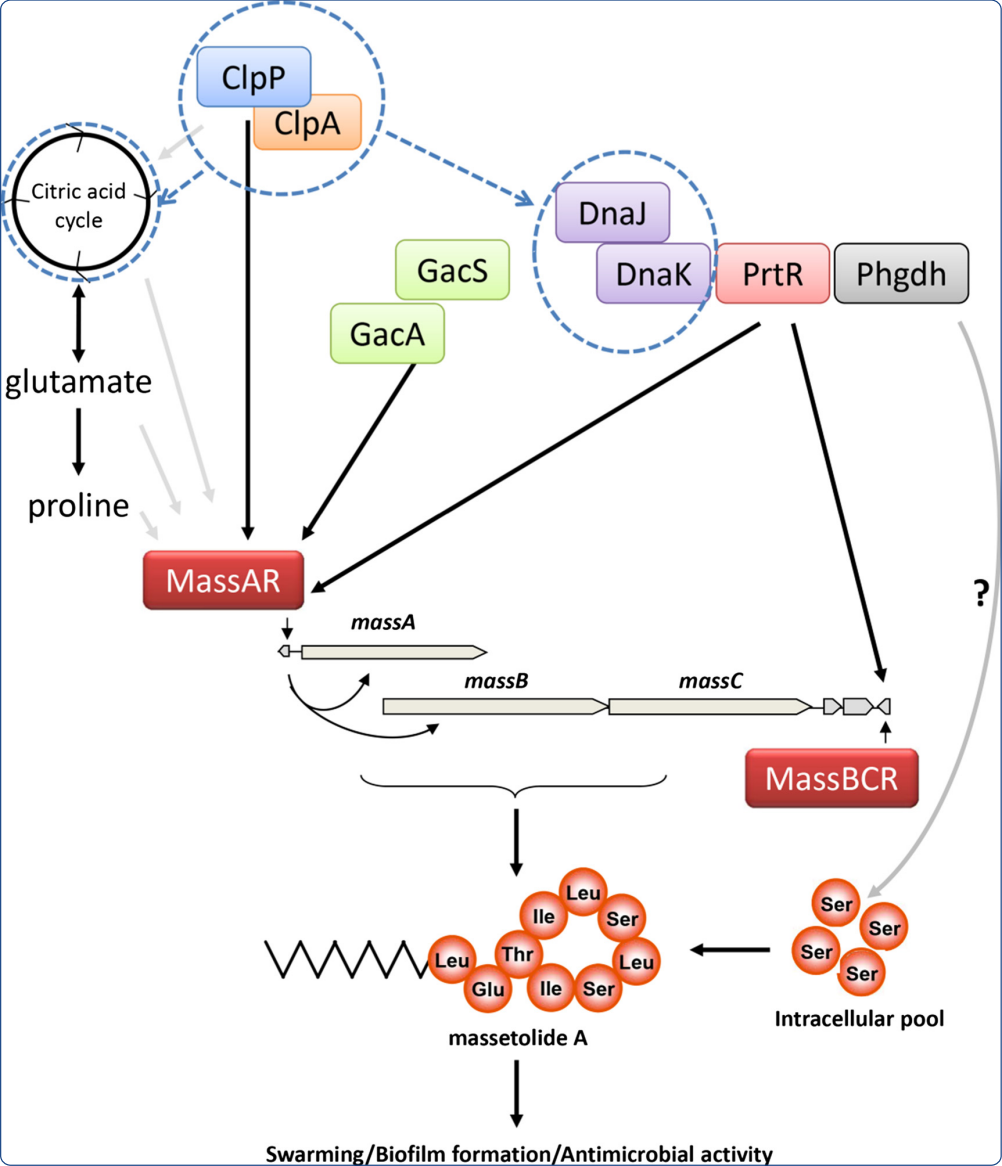
**Proposed model for the genetic regulation of massetolide biosynthesis in** ***P. fluorescens*** **strain SS101.** The darkly shaded arrows are based on experimental data obtained earlier [10] and in this study; the lightly shaded arrows are hypothetical and not based on experimental data. The blue dashed arrows and circles represent translational regulation whereas the other arrows represent transcriptional regulation.

In both *clpA* and *clpP* mutants, 32 and 39 proteins were up- and down-regulated, respectively (Table 2, Additional file 2: Table S5). The most up-regulated was CspD (PflSS101_3195), a gene encoding one of the cold shock protein CspA family members in *E. coli.* CspD is known to be induced by nutritional deprivation [25]. Moreover, the response regulator CbrB and the transcriptional regulator GntR were up-regulated in both mutants. The CbrA-CbrB two-component system is known to control the utilization of different carbon and nitrogen sources in *P. aeruginosa* [26] and affects chemotaxis, stress tolerance and biofilm development in *Pseudomonas putida* [27]. GntR is a transcriptional regulator that controls antibiotic production in both *Streptomyces* and *Serratia* [28,29]. None of these proteins and their corresponding genes were found in genome-wide screening for massetolide-deficient mutants, except DnaK [10]. In our proteome analyses, the DnaK protein was found at higher concentrations in the *clpP* mutant and its chaperon DnaJ protein was up-regulated in both *clpA* and *clpP* mutants. Given that DnaK and DnaJ also regulate putisolvin biosynthesis in *P. putida* [30], our results suggest that ClpAP regulates LP biosynthesis in multiple *Pseudomonas* species at least in part, via DnaK and DnaJ (Figure 4).

**Table 2:** **Up-regulated proteins in both** ***clpA*** **and** ***clpP*** **mutants of** ***Pseudomonas fluorescens*** **SS101**

**Locustag**	**Gene**	**Gene descriptions**	**∆** ***clpA*** **/SS101**	**∆** ***clpP*** **/SS101**
PflSS101_0002	*dnaN*	DNA polymerase III, beta subunit	1.3	1.3
PflSS101_0021	*qor*	NADPH_quinone reductase	1.25	1.6
PflSS101_0364	*secB*	protein-export chaperone SecB	1.34	1.42
PflSS101_0509	*thiC*	thiamine biosynthesis protein ThiC	1.33	1.28
PflSS101_0546	*rnr*	ribonuclease R	1.27	1.27
PflSS101_0920	*hisC_1*	histidinol-phosphate transaminase	1.3	1.2
PflSS101_0926	*mqo_1*	malate_quinone-oxidoreductase	1.32	1.21
PflSS101_1161	*argG*	argininosuccinate synthase	1.3	1.2
PflSS101_1203		TIGR00730 family protein	1.22	1.22
PflSS101_1209	*fpr_2*	ferredoxin--NADP+ reductase	1.28	1.24
PflSS101_1348	*fabD*	acyl-carrier-protein S-malonyltransferase	1.26	1.32
PflSS101_1554		LamB_YcsF family protein	1.25	1.27
PflSS101_1626		short-chain alcohol dehydrogenase family protein	1.53	1.23
PflSS101_1652	*cmk*	cytidylate kinase	1.35	1.36
PflSS101_1729		3-deoxy-7-phosphoheptulonate synthase	1.28	2
PflSS101_2196		AP endonuclease, family 2	1.65	2.12
PflSS101_3195		cold shock domain protein CspD	2.14	3.15
PflSS101_3348	*bkdA2*	2-oxoisovalerate dehydrogenase E1 component, beta subunit	1.26	1.23
PflSS101_3776		flagellin domain protein	1.21	2.14
PflSS101_3786	*phhA*	phenylalanine-4-hydroxylase	1.24	1.81
PflSS101_3936		transcriptional regulator, GntR family	1.25	1.35
PflSS101_4181		conserved hypothetical protein	1.2	1.2
PflSS101_4298	*tolB*	Tol-Pal system beta propeller repeat protein TolB	1.33	1.29
PflSS101_4316		PF04461 family protein	1.21	1.55
PflSS101_4394	*thrC*	threonine synthase	1.29	1.43
PflSS101_4600	*cbrB*	two-component response regulator CbrB	1.25	1.5
PflSS101_4631	*dapB*	dihydrodipicolinate reductase	1.5	1.55
PflSS101_4632	*dnaJ*	chaperone protein DnaJ	1.26	1.22
PflSS101_4676		conserved hypothetical protein	1.31	1.21
PflSS101_4945	*rpsU*	ribosomal protein S21	1.25	1.25
PflSS101_5275	*rnk*	regulator of nucleoside diphosphate kinase	1.52	1.65
PflSS101_5280	*lysA*	diaminopimelate decarboxylase	1.23	1.27

### TCA cycle proteins were expressed differently in both *clpP* and *clpA* mutants

Our proteome analyses also revealed that several proteins from the TCA cycle were differentially expressed in both the *clpA* and the *clpP* mutants (Additional file 1: Figure S3). Five proteins were down-regulated and two were up-regulated in the *clpA* mutant. Similar numbers of down-regulated (6) and up-regulated (2) proteins were found in the *clpP* mutant (Additional file 1: Figure S3). In the TCA cycle, PckA (PflSS101_0285) encodes phosphoenolpyruvate carboxykinase ATP and transfers oxaloacetate to phosphoenolpyruvate. This protein was 1.20 up- and 1.47 down-regulated in the *clpA* and *clpP* mutants, respectively. Mqo_1 (PflSS101_0926), a malate quinone oxidoreductase, was up-regulated in both mutants. Malate quinone oxidoreductase is known to be essential for growth on ethanol or acetate in *Pseudomonas aeruginosa* [31]. It is also required for virulence of *Pseudomonas syringae* pv. tomato strain DC3000 on *Arabidopsis thaliana* [32]. Its function in *P. fluorescens* SS101, however, is not yet known.

## Conclusions

ClpA is a chaperon protein that is highly conserved in bacteria and eukaryotes [33,34]. Together with the serine protease ClpP, it plays an important role in intracellular refolding and degradation of proteins, an essential process for the viability and growth of cells. In this study, we cloned and sequenced *clpA* from the plant growth-promoting bacterium *P. fluorescens* strain SS101 and showed that *clpA* plays an important role in the regulation of massetolide biosynthesis. The combined results of the transcriptomic and proteomic analyses suggest that the ClpAP complex regulates massetolide biosynthesis via the pathway-specific LuxR-type transcriptional regulator MassAR, the heat shock proteins DnaK and DnaJ and via proteins involved in the TCA cycle. These findings extend our previous regulatory model for LP biosynthesis in *P. fluorescens* SS101 (Figure 4) which, to a large extent, may also apply to the regulatory networks of LP biosynthesis in other *Pseudomonas* species and strains.

## Methods

### Bacterial strains and culture conditions

*P. fluorescens* strain SS101 and its *clpP* and *clpA* mutants were cultured in King’s medium B (KB) broth at 25°C. The *clpA* and *clpP* mutants were obtained in our previous studies [10,11]. *Escherichia coli* strain DH5α was the host for the plasmids used for genetic complementation. *E. coli* strains were grown on Luria-Bertani (LB) plates or in LB broth amended with the appropriate antibiotics.

### Identification of the *clpA* cluster

*clpA* was identified by partial sequencing of the regions flanking the plasposon insertion as described by Song et al. [10]. The complete flanking regions of *clpA* were obtained from the genome sequence of *P. fluorescens* SS101 [35]. Open reading frames (ORFs) were identified with the Softberry FGENESB program (http://www.softberry.com/berry.phtml). The ORFs were analyzed using BlastX in the NCBI database and Pseudomonas.com (http://pseudomonas.com). For genetic complementation, the pME6031-*clpA* construct was generated according to methods described previously [11]. Briefly, a 2,870-bp fragment, including the promoter and terminator, was subcloned into the shuttle vector pME6031 and transformed into. *E. coli* DH5α. The pME6031-*clpA* construct was subsequently electroporated into the *clpA* plasposon mutant of *P. fluorescens* SS101. Transformed cells were plated on KB supplemented with tetracycline (25 μg/ml), and the presence of pME6031-*clpA* was verified by PCR analysis with primers specific for pME6031.

### Lipopeptide extraction and RP-HPLC separation

Massetolide extractions and RP-HPLC analysis were performed as described earlier [8,10,11]. Briefly, *Pseudomonas* strains were grown on Pseudomonas isolation agar plates (Pseudomonas agar 38 g/L, glycerol 10 g/L) for 48 h at 25°C. The cells were suspended in sterile de-mineralized water (~40 ml per plate), transferred to 50 mL tubes, shaken vigorously for 2 min and then centrifuged (30 min, 5292 g, 4°C). The culture supernatant was transferred to a new tube and acidified to pH 2.0 with 9% HCl. The precipitate was recovered by centrifugation (30 min, 5292 g, 4°C) and washed three times with acidified dH_2_O (pH 2.0). It was then resuspended in 5 mL dH_2_O and the pH adjusted to 8.0 with 0.2 M NaOH until complete dissolution. The solution was centrifuged (30 min, 5292 g, 4°C) and the supernatant transferred to a new tube, subjected to lyophilisation and RP-HPLC analysis according to methods described previously [36].

### Swarming motility

Swarming motility assays of the wild-type and mutants strains were performed as described earlier [10]. Swarming motility of the wild-type SS101 strain and the mutants was assessed on soft [0.6% wt/vol] standard succinate agar medium (SSM) consisting of 32.8 mM K_2_HPO_4_, 22 mM KH_2_PO_4_, 7.6 mM (NH_4_)_2_SO_4_, 0.8 mM MgSO_4_, and 34 mM succinic acid. The pH of the medium was adjusted to 7 with NaOH. Cells from overnight cultures of the wild-type and mutant strains were washed three times with 0.9% NaCl, and 5 μL of the washed cell suspensions (1 × 10^10^ cells/ml) was spot inoculated in the centre of the soft SSM agar plate and incubated for 48–72 h at 25°C.

### Transcriptome analysis

The wild-type SS101 strain and the *clpA* and *clpP* mutants were grown in KB broth in 24-well plates, and harvested for RNA isolation at an OD_600nm_ = 0.6. For each strain, three biological replicates were used. Total RNA was extracted with Trizol reagent (Invitrogen) and further purified with the NucleoSpin RNA kit. A tiling microarray for *P. fluorescens* SS101 was developed by the Dutch Genomics Service & Support Provider, University of Amsterdam (UvA, Amsterdam, the Netherlands). In total, 134,276 probes (60-mer) were designed with, in general, a gap of 32 nucleotides between adjacent probes on the same strand and an overlap of 14 nucleotides for both strands. In addition, 5,000 custom negative control probes were hybridized and used as an internal control to validate the designed probes in a CGH experiment of 4 arrays. Probes were annotated and assembled into probe sets for known genes based on location information retrieved from the Pathosystems Resource Integration Center (PATRIC, http://patricbrc.org). Probes outside of known gene sequences were labeled as InterGenic Region (IGR). cDNA labelling was conducted as described previously [37]. Briefly, cDNA was synthesized in presence of Cy3-dUTP (Cy3) for the test samples and with Cy5-dUTP (Cy5) for the common reference. The common reference consisted of an equimolar pool of the test samples (3 μg per sample). 5 μg of total RNA per reaction was used and yielded 1.5-2.5 μg cDNA for each sample with larger than 16 pmol of Cy3 or Cy5 dye per microgram. Hybridizations were performed as described elsewhere [38]. Slides were washed according to the procedures described in the Nimblegen Arrays User’s Guide - Gene Expression Arrays Version 5.0 and scanned in an ozone-free room with an Agilent DNA microarray scanner G2565CA (Agilent Technologies). Feature extraction was performed with NimbleScan v2.5 (Roche Nimblegen). Data pre-processing consisted of log_2_-transformation of the raw probe-intensity data, followed by a within slide Lowess normalization. Thus normalized sample (Cy3) channel intensities were summarized into probe sets values and normalized between arrays using the RMA (Robust Multi-Array Analysis) algorithm [39]. Analysis of the gene expression data was conducted using the Arraystar software. All results described were found to be significant using a false discovery rate of less than 5%.

### Proteome analysis

The wild-type SS101 strain and the *clpA* and *clpP* mutants were grown in KB broth in 24-well plates, and cells were harvested for protein extraction at an OD_600nm_ = 0.6. Three biological replicates were used for each strain. The cells were harvested by centrifugation and resuspended in 15 mL ice-cold 1 x PSB buffer containing the protease Inhibitor Cocktail from Sigma-Aldrich, as instructed by the manufacturer. The following steps were performed at 4°C. The cells were disrupted twice in a French pressure cell press (SLM Instruments Inc) at 14,000 psi and centrifuged for 30 min at 47,000 g. Protein concentration was determined using the Bradford assay followed by iTRAQ labeling in a 4-plex experiment according to the manufacturer’s protocol (AB Sciex Pte. Ltd). Briefly, 100 μg of protein in 100–400 μL were successively reduced in the presence of 1 μL TCEP (tris(2-carboxyethyl)phosphine), alkylated using 2 μL 85 mM iodoacetamide, and hydrolyzed with 2.5 μg trypsin. A further addition of 2.5 μg trypsin 1 h after the initial addition of the protease was performed prior to an overnight incubation. Each of the reaction mixtures was then freeze-dried, redissolved in 100 μL 125 mM TEAB (triethylammonium bicarbonate) in 75% ethanol and transferred to one vial of iTRAQ reagent (4-plex, 114–117). After 1 h incubation, 100 μL of H_2_0 was added followed by 15 min incubation in order to hydrolyze the excess of iTRAQ reagent. The resulting samples were pooled together and desalted using SepPak C18 cartridges (Waters Corporation). The pooled samples (800 μL) were diluted to 3.6 mL in 0.1% formic acid (FA) and loaded onto pre-wetted (95% acetonitrile (ACN) containing 0.1% FA) and equilibrated (0.1% FA) cartridges. After washing the loaded cartridges 5 times with 1 mL 0.1% FA, elution was performed in 1 mL 50% ACN/0.1% FA followed by 95% ACN/0.1% FA. Eluates were combined and evaporated to dryness.

The evaporated iTRAQ-labeled samples were resolubilized (10 μL) in the sample loading buffer (5 mM ammonium acetate containing 5% ACN) and injected (4.9 μL) using the partial loop mode on a liquid chromatograph (nanoAcquity UPLC system, Waters Corporation) plumbed for two-pump trapping and two-dimensional strong-cation exchange and reversed-phase (SCX-RP) separation. Salt plugs (10, 20, 30, 40, 50, 80, 150, 200 mM ammonium acetate in 5% ACN, followed by 200 mM in 30% ACN and 350 mM in 50% ACN) were injected using the full loop mode. Sample and salt plugs were loaded in trap mode (SCXtrap-C18trap-waste) onto the SCXtrap column (18x20mm, 5 μm particle size, P/N 186003507) using the sample and loading buffer for 10 min at 5 μL/min. Subsequently, an analytical separation was performed in analytical mode (C18trap-C18Analytical-ESI source) at 400 nL/min with the following consecutive steps and gradient: 1% B (100% ACN, 0.1% FA) (0–1 min); 1–40% B (1–50 min); 40-60% B (50–65 min); 60-85% B (65–66 min); 85% B (66–70 min); 85-1% B (70–71 min).

The gradient flow from the nanoAcquity was delivered into the Nano ESI ion source of a Xevo Q-TOF mass spectrometer from Waters Corporation (source voltage 4 kV; source temperature 80°C; cone voltage 35 V; cone gas flow 20 L/h; nano flow gas 0.8 bar). Data were acquired in data dependent mode with one full scan (350–1400 m/z) followed by maximum 5 MS/MS scans (50–1800 m/z) on doubly and triply charged peptides only. External TOF mass calibration was performed prior to the UPLC-MS analysis. This was obtained by direct infusion of a solution containing 2 g/L sodium iodide in 50% isopropanol, and data acquisition in TOF-MS mode over the *m/z* range 50–2000.

#### Proteome data analysis

Raw data files were treated using the trans-proteomic pipeline (TPP) software package for proteomic data analysis supplied by the Seattle Proteome Centre [40]. The processing of data through the TPP modules was automated by in-house java-based software. Initially, raw files were converted into uncentroided mzXML files using MSConvert. Before search all data was centroided and processed to only keep the top 100 peaks in each fragment spectra. Centroided data was then analysed using X!tandem with native scoring. Search hits from each individual replicate were assigned probabilities using Peptide Prophet [41] utilizing the semi-parametric model, at this stage each technical-replicate was assigned a unique experiment ID to allow iProphet [42] to utilize the number of replicate experiments model. Libra (TPP module) was then used to extract iTRAQ reporter ion signals from the uncentroided data, in each replicate the four different iTRAQ reporter channels were normalized to account for 25% of the total signal.

Each set of technical replicates were then combined into a single output pep.xml using iProphet [43] and final protein lists were assembled using Protein Prophet [44] and Libra was used to calculate iTRAQ protein ratios. Parameters used for analysis were as follows; X!tandem searches were ran against the P. fluorescens SS101 amino acid sequence database, concatenated to its own reversed sequences for use as decoy hits. Searches used trypsin specificity, a precursor ion tolerance of 50 ppm, a fragment monoisotopic tolerance of 0.4 Da and the following post-translational modifications were assigned; fixed carbamidomethyl cystein, fixed iTRAQ (N-term), fixed iTRAQ (K), variable oxidation (M), variable iTRAQ (Y), variable phosphorylation (S/T). Libra protein ratios were extracted using intensity weighted average, using normalization by sum of reagent profiles, minimum reporter ion intensity of 20 and a reporter ion mass tolerance of 0.05.

## Additional files

Additional file 1: Figure S1. Differential gene transcription between the wild-type *P. fluorescens* SS101 strain and the *clpA* (A) or *clpP* (B) mutant at exponential phase (OD600 = 0.6), assessed by microarray analyses. The transcription chart shows log2-based fold changes of transcripts of *clpA* or *clpP* mutant compared to the wild-type strain SS101. Each dot in the chart represents each of the 5374 annotated genes in the SS101 genome with the x-axis showing gene order, and the y-axis showing the log2 of relative transcripts abundance for each gene in the *clpA* or *clpP* mutant compared to the wild-type strain SS101. Gene clusters whose members are discussed in the main text are shown. **Figure S2.** Differential protein expression between wild-type *P. fluorescens* SS101 and the *clpA* (A) or the *clpP* (B) mutant at exponential phase (OD600 = 0.6), assessed using isobaric tag labeling for relative and absolute quantitation (iTRAQ) experiments. The expression chart shows fold changes of protein expression in the *clpA* or *clpP* mutant compared to the wild-type strain SS101. Each dot in the chart represents the 200 and 223 proteins that significantly accumulated in the *clpA* and *clpP* mutants, respectively. The x-axis shows gene order and the y-axis shows fold changes. **Figure S3.** TCA cycle pathway of *P. fluorescens* SS101 (adjusted from KEGG with *P. fluorescens* A506, the most related strain of SS101). Red boxes indicate up-regulation; green boxes indicate down-regulation; empty boxes stand for “not detected”. The left and right boxes stand for protein expression in the *clpA* and *clpP* mutants, respectively. Additional file 2: Table S1. Whole genome transcriptome analysis of ∆*clpA*/wt. **Table S2.** Whole genome transcriptome analysis of ∆*clpP*/wt. **Table S3.** Whole genome proteome analysis of ∆*clpA*/wt. **Table S4.** Whole genome proteome analysis of ∆*clpP*/wt. **Table S5.** Down-regulated proteins in both *clpA* and *clpP* mutants of *Pseudomonas fluorescens* SS101.
