# Supplementary figures and images for: Lipopeptide biosynthesis in Pseudomonas fluorescens is regulated by the protease complex ClpAP
Source: BMC Microbiol. 2015 Feb 14;15:29. doi: 10.1186/s12866-015-0367-y (PMC4332742; doi:10.1186/s12866-015-0367-y)

Figure S1

A

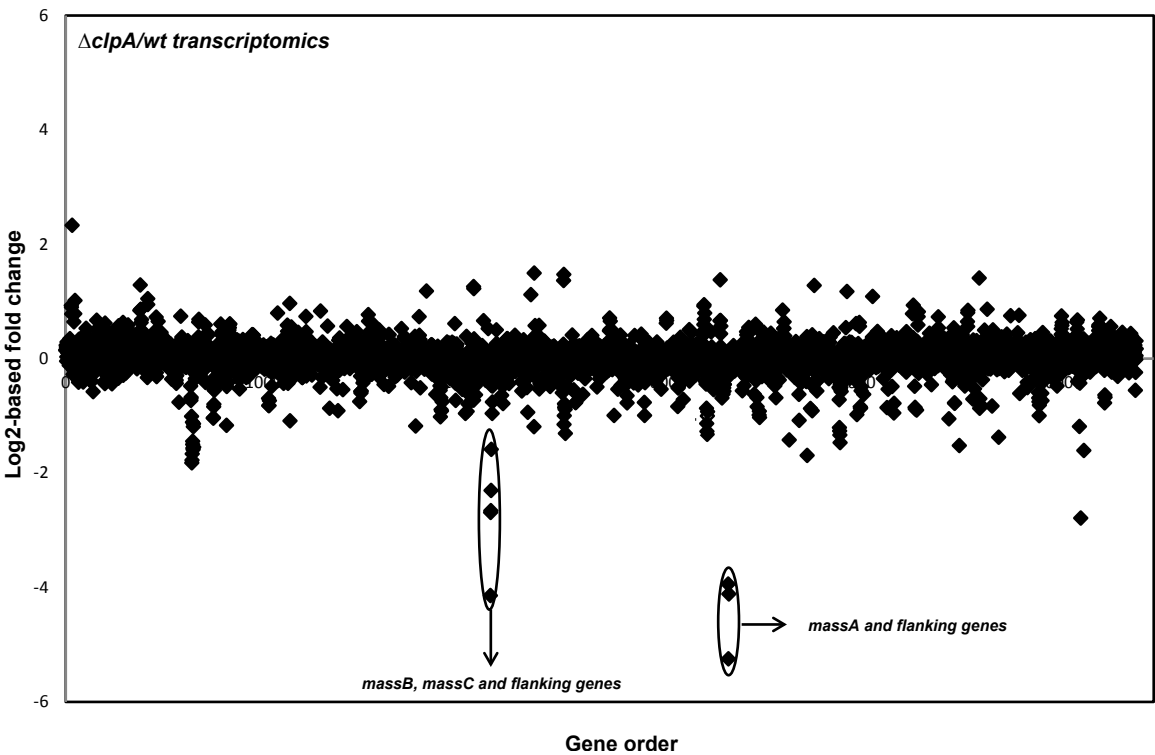

B

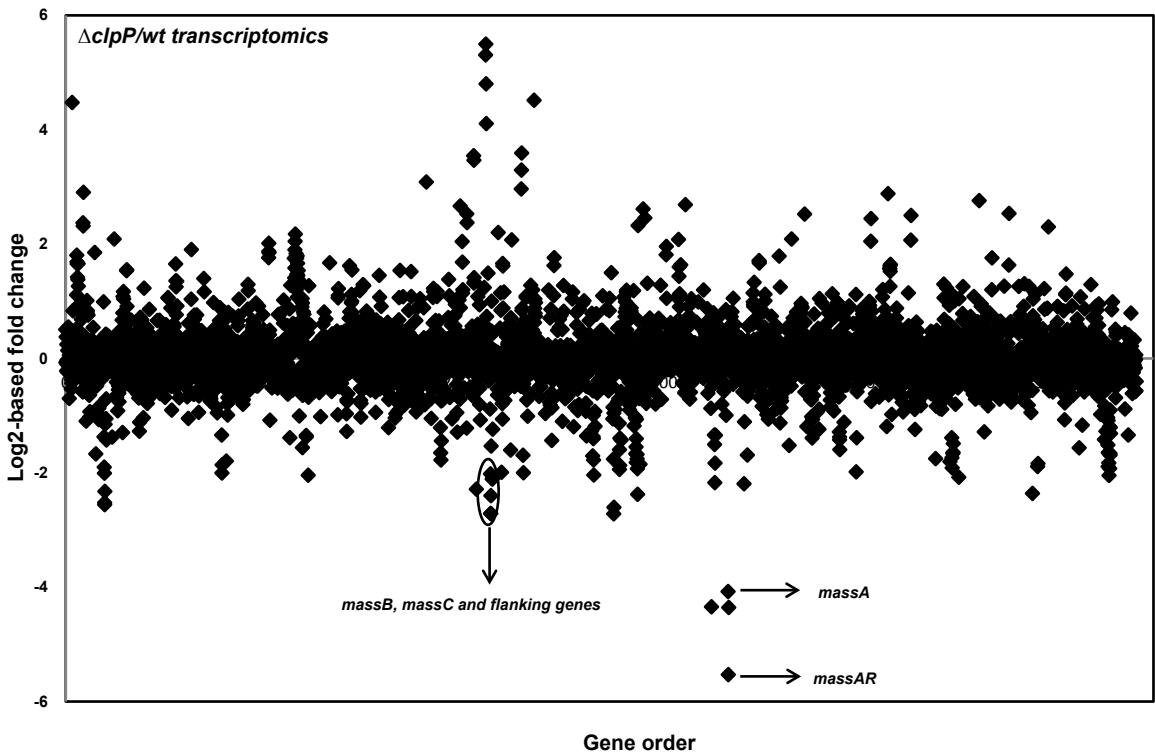

Figure S2

A

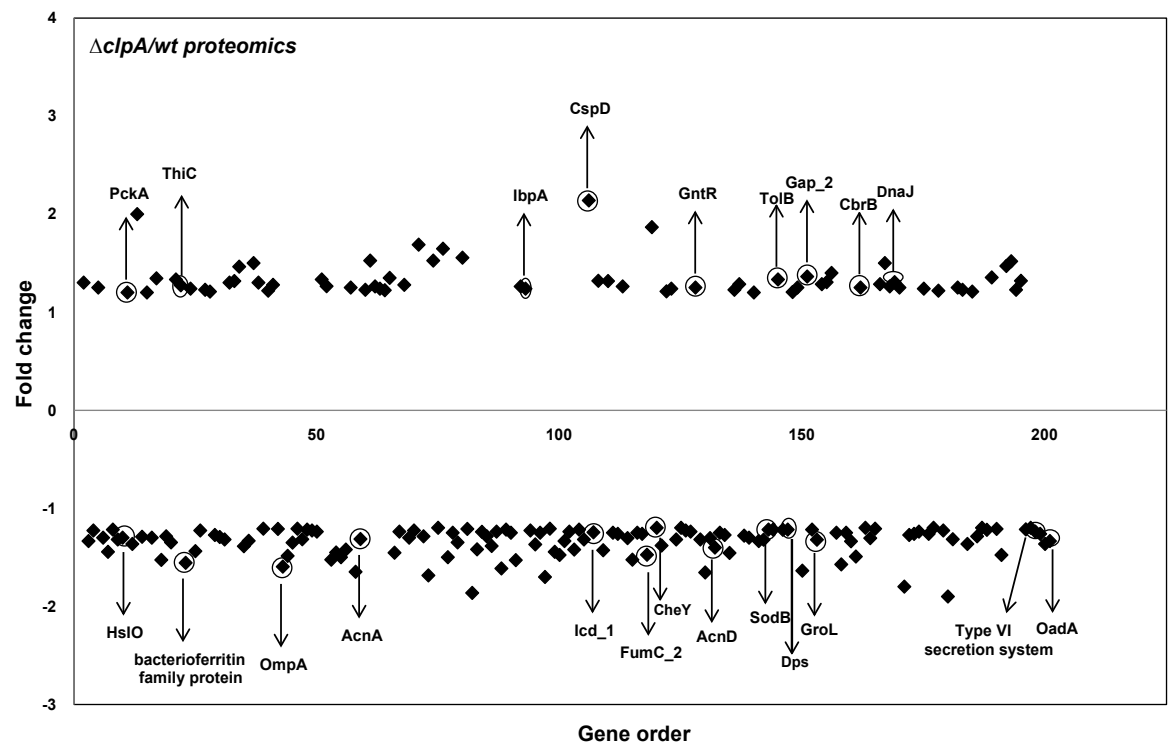

B

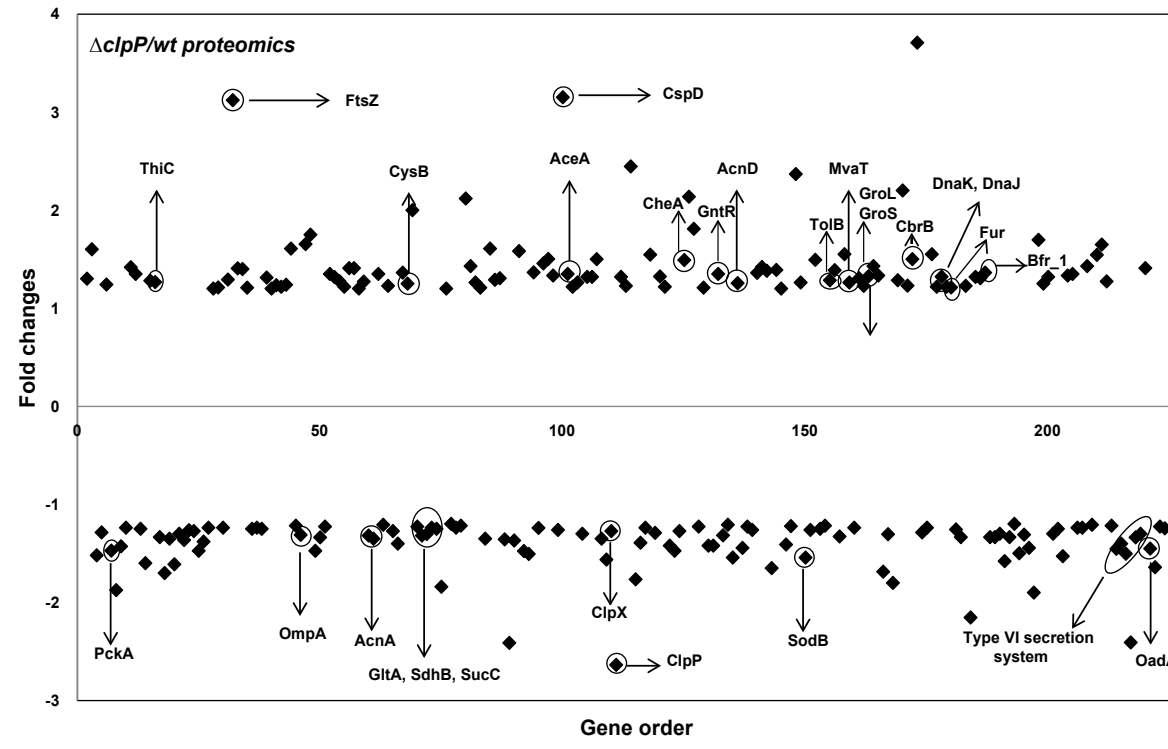

Figure S3

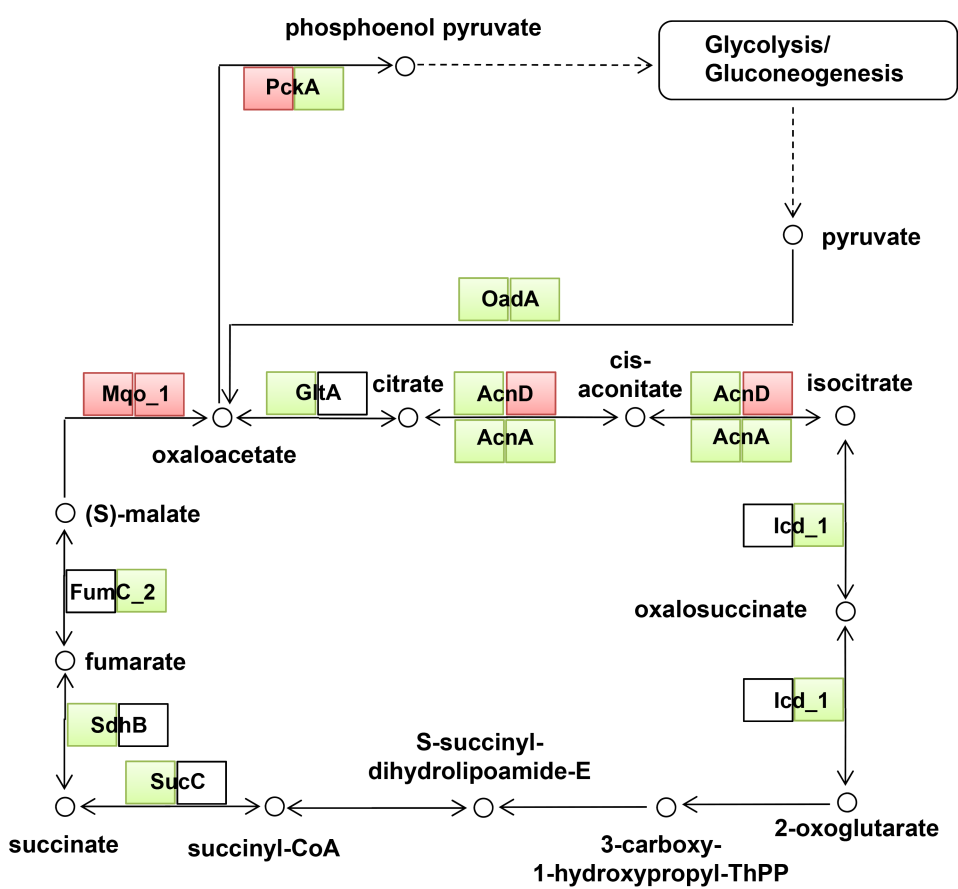

Supplement: Additional file 1: Figure S1. — Differential gene transcription between the wild-type P. fluorescens SS101 strain and the clpA (A) or clpP (B) mutant at exponential phase (OD600 = 0.6), assessed by microarray analyses. The transcription chart shows log2-based fold changes of transcripts of clpA or clpP mutant compared to the wild-type strain SS101. Each dot in the chart represents each of the 5374 annotated genes in the SS101 genome with the x-axis showing gene order, and the y-axis showing the log2 of relative transcripts abundance for each gene in the clpA or clpP mutant compared to the wild-type strain SS101. Gene clusters whose members are discussed in the main text are shown. Figure S2. Differential protein expression between wild-type P. fluorescens SS101 and the clpA (A) or the clpP (B) mutant at exponential phase (OD600 = 0.6), assessed using isobaric tag labeling for relative and absolute quantitation (iTRAQ) experiments. The expression chart shows fold changes of protein expression in the clpA or clpP mutant compared to the wild-type strain SS101. Each dot in the chart represents the 200 and 223 proteins that significantly accumulated in the clpA and clpP mutants, respectively. The x-axis shows gene order and the y-axis shows fold changes. Figure S3. TCA cycle pathway of P. fluorescens SS101 (adjusted from KEGG with P. fluorescens A506, the most related strain of SS101). Red boxes indicate up-regulation; green boxes indicate down-regulation; empty boxes stand for “not detected”. The left and right boxes stand for protein expression in the clpA and clpP mutants, respectively. [file 12866_2015_367_MOESM1_ESM.pdf]
